# How does evolution work in superabundant microbes?

**DOI:** 10.1016/j.tim.2024.01.009

**Published:** 2024-02-14

**Authors:** Dmitry A. Filatov, Mark Kirkpatrick

**Affiliations:** 1 Department of Biology, University of Oxford, Oxford, OX1 3RB, United Kingdom; 2 Department of Integrative Biology, University of Texas, Austin TX 78712 USA

**Keywords:** evolution, population size, genetic diversity, marine plankton, superabundant microbes, evolutionary genetic methods

## Abstract

Marine phytoplankton play crucial roles in the Earth’s ecological, chemical, and geological processes. It is responsible for about half of global primary production and drives the ocean biological carbon pump. Understanding how plankton species may adapt to the Earth’s rapidly changing environments is evidently an urgent priority. This problem requires evolutionary genetic approaches as evolution occurs at the level of allele frequency change within populations driven by genetic drift and natural selection (microevolution). Plankters such as the coccolithophore *Gephyrocapsa huxleyi* and the cyanobacterium *Prochlorococcus “marinus”* are among Earth’s most abundant organisms. In this Opinion paper we discuss how evolution in astronomically large populations of superabundant microbes (SAMs) may act fundamentally differently than it does in the populations of more modest size found in well-studied organisms. This offers exciting opportunities to study evolution in the conditions that have yet to be explored and also leads to unique challenges. Exploring these opportunities and challenges is the goal of this paper.

## Why is understanding evolution in SAMs so important?

The importance of superabundant microbes, such as marine phytoplankton, is difficult to underestimate – they form the basis of the food chain and are responsible for about half of newly produced organic matter on the planet and half of the oxygen that we breathe [1]. Understanding how evolutionary processes operate in marine plankton is critical for predicting their ability to spread (e.g. polar-wards [2]), adapt to ever-changing environments (e.g. being constantly advected by currents [2–4]), and their resilience to rapid global environmental change [5,6]. Yet, surprisingly little is known about population genetic processes underpinning evolution of these microscopic but hugely important organisms [7,8]. Evolutionary genetic processes have largely been studied for organisms whose population sizes are relatively small (*e*.*g*. primates) to relatively large (*e*.*g*. Drosophila). The population sizes of SAMs, however, are yet larger by many orders of magnitude. For example the census sizes of marine phytoplankton coccolithophore *Gephyrocapsa* (ex- *Emiliania*) *huxleyi* and cyanobacterium *Prochlorococcus* are of the order 10^22^ [9] and 10^28^ [10] cells, respectively – truly astronomical values that are comparable to the number of stars in the Universe. During its seasonal blooms, *G. huxleyi* can be so abundant as to be visible from space despite the microscopic cell size (~5μ). Below we discuss why evolutionary processes in these vast populations may operate differently compared to much smaller populations [11–13].

Over the last 50 years, geneticists have developed an extensive statistical toolbox to study evolution at the levels of genes and populations (e.g. [14,15]). These powerful evolutionary approaches can be informative about many aspects of biology and evolution in marine microbes, such as presence/absence of sexual reproduction in non-model organisms [16–18], genome evolution [19], including the role of accessory genes in pangenomes of microorganisms [20], adaptation to local environmental conditions [21–23], and speciation in marine microorganisms [13,24]. Evolutionary genetics could also be used to infer past environmental conditions [25] and to study the evolutionary processes underpinning patterns seen in the fossil record. For example, a recent paleontological study reported that climatic changes associated with variation in the Earth’s orbit are driving cyclic changes in morphology in the dominant Cenozoic Noelaerhabdaceae family of coccolithophores [26]. An evolutionary genetic analysis provided complementary insight: oscillations in size and abundance of coccolithophore fossils were caused by consecutive radiations and extinctions of species rather than variation in the abundance of species with different cell sizes [27].

Evolutionary genetic reconstructions of past population size changes of marine phytoplankton species (e.g. [24,27]) may be important for inferences in ocean biogeochemistry; *e*.*g*. foraminifera are widely used as proxies for ocean surface temperatures [28] and reconstructing their abundance through time would be valuable for paleoclimatic modelling. Global warming has raised concerns about the resilience of marine phytoplankton to rising sea temperature, ocean acidification [6] and plankton feedback to climate change [29]. The studies of phytoplankton performance in different conditions [30,31], revealed that strains of the same species isolated from different locations are well adapted to local environmental conditions [32,33], which implies surprisingly rapid adaptation, given they are constantly advected by currents [3,4]. Without understanding the evolutionary genetic processes underpinning this adaptation it is difficult to predict the ability of phytoplankton species to adapt to rapidly changing environment [34] and to affect global climate [35].

In this paper we explore how the extreme population sizes of superabundant microbes may cause them to evolve in unusual, perhaps unique, ways. We discuss which of the standard methods of molecular evolution are applicable to SAMs and which can go wrong or need modifications to accommodate the unusual biology of these organisms. We identify the methods that appear to be unsuitable to SAMs, and suggest they are priorities for the development of new theory and statistics.

## Why does population size matter?

### The role of random genetic drift and selection

The role that population size and genetic drift play in evolution (Box 1) was first worked out in the early 20^th^ Century by Fisher, Wright, and Haldane; further important advances were later made by Kimura [36]. Their theory shows that for genomic sites that are free from selection (“neutral”), nucleotide diversity (π) is proportional to the “effective size” of the population, *N*_e_, and the mutation rate, μ (Equation 1 in Box 1). When an estimate of μ is available, this simple relation can be used to estimate *N*_e_ from molecular data. At 4-fold degenerate sites (thought to be the most neutral of sites in the genome [37]), nucleotide diversity in the coccolithophore *G. huxleyi* is π ~ 0.005 [38], while in the cyanobacterium *Prochlorochoccus* it is π = 0.005 – 0.041 [10]. Estimates for their mutation rates are respectively μ = 5x10^-10^ – 6x10^-10^ [39] and μ = 2x10^-10^ – 5x10^-10^ [10] per site per cell division. From Eq. (1), these data imply that the effective population sizes for these microbes are of the order *N*_e_ = 10^6^ – 10^7^. Similar estimates come from other abundant marine plankton: *N*_*e*_ ~ 10^7^ for the unicellular green algae *Osteococcus taurii* [18] and the dinoflagellate *Alexandrium ostenfeldii* [40]. What is so striking about these results is that they are wildly at variance with direct observations of these microbe’s population sizes: these numbers of cells can be found in just 500 ml of seawater.

**Figure 1:**
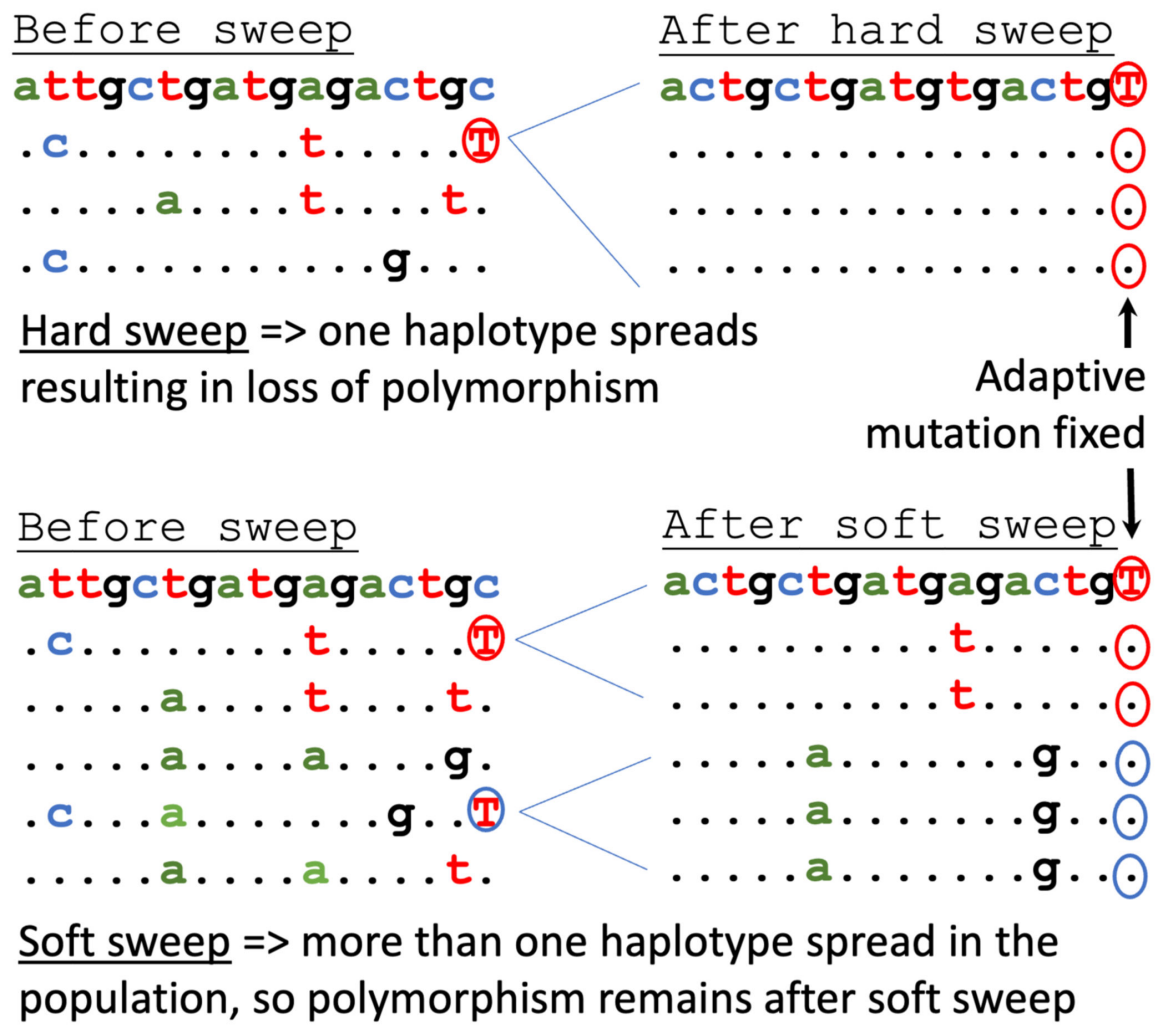
The effects of adaptation on genetic diversity. Spread and fixation of a single adaptive mutation (circled) results in loss of genetic diversity – a so-called "selective sweep" [71] or "hard sweep", as shown in the two upper panels. On the other hand, if the adaptive mutation arises more than once independently, their spread does not eliminate all genetic variation in that region of the genome, making adaptation difficult to detect – a so-called "soft sweep" [84], shown in the two bottom panels. Each panel shows a sequence alignment with dots standing for the same nucleotide as in top row to highlight the nucleotide polymorphisms. Red and blue circles around the adaptive allele show the same mutation that arose independently in different haplotypes.

The huge disparities between the expected and observed nucleotide diversities in these species represent extreme instances of a general phenomenon called “Lewontin’s paradox” [38] – the unexpectedly low genetic diversities in very large populations that was described by R.C. Lewontin back in 1974 [41]. A variety of hypotheses have been offered to account for this paradox [42], but a general consensus has yet to be reached [43]. Some marine microbes have largely clonal (asexual) reproduction, which could, at least partly, account for reduced genetic diversity [16], because the loss of polymorphism due to genetic hitch-hiking, such as selective sweeps (Figure 1), is much more extensive in clonal populations. However, this explanation does not apply to species where sex and recombination are frequent, such as diatoms. A leading hypothesis is that genetic diversity is reduced by population bottlenecks [42]. Indeed, populations of marine plankton tend to follow bloom-and-bust dynamics. Following a bottleneck, π is slow to grow back to its equilibrium value, and so *N*_e_ estimated from π is expected to be close to the population size during the bottleneck for a long period afterwards [42]. It is hard to imagine, however, that even during a bottleneck the total number of cells of a globally distributed plankton species such as *G. huxleyi* was so low as only ~10 million. Any population subdivision, e.g. caused by distinct environmental conditions [44] or partial isolation between water bodies [45], would only increase overall genetic diversity (and *N*_e_ estimated from species-wide π) due to divergence between sub-populations, limiting the effect of bottlenecks on genetic diversity.

We propose an explanation for Lewontin’s paradox in SAM that is often neglected in the literature. The relative contributions of drift and natural selection to the evolution of a mutation is determined by the population’s effective size (*N*_e_) and the mutation’s “selection coefficient”, symbolised by *s* (Box 2). A key point is that when |*N*_e_ *s*| < 1, the mutation is expected to evolve as if it is neutral, even if it is not (*i*.*e*. |*s*| > 0). For SAMs such as *G. huxleyi* and *Prochlorococcus*, it is plausible that the true *N*_e_ (rather than *N*_e_ estimated from π) is so large that very few or even no mutations have fitness effects sufficiently small that |*N*_e_ *s*| < 1. That is, virtually all mutations are deleterious and quickly removed by natural selection, causing genetic diversity to be much smaller than expected from Eq. (1). Occasionally arising adaptive mutations also do not contribute much to polymorphism because they spread and fix quickly. This may be sufficient to account for the extreme examples of Lewontin’s paradox in SAMs.

Putting this discussion into an historical context: the hypothesis that all mutations experience selection and that most are deleterious harkens back to “classical” or “panselectionist” view during the famous debate between those who favoured Kimura’s neutral model of molecular evolution and those who did not [41]. While a modified version of Kimura’s model – the “nearly neutral theory” [46] – is widely accepted for evolutionary genetic processes in populations of almost all organisms (eukaryotes and prokaryotes alike), the panselectionist view may be more suitable for the astronomical population sizes of SAMs, such as marine phytoplankton.

### The input of beneficial mutations

A second crucial role that population size plays in evolution is to modulate the number of beneficial mutations that enter a population. As the census size of the population (*N*_c_) grows, the total number of mutations entering the population each generation (= *N*_c_ μ) increases. This effect allows larger populations to adapt more quickly, *e*.*g*. to changing environments. *G. huxleyi* has a per-site mutation rate of ~5 x 10^-10^ [39]. Its census population size is conservatively estimated at *N*_c_ = 10^22^ cells [9]. These divide at a rate of about once a day in lab culture [39], and likely at a much lower rate when the environmental conditions are not ideal. Say that cells in natural populations divide once a week on average. Then every base pair in the genome mutates somewhere in the population about 10^11^ times per day (and even more frequently when conditions are favourable, *e*.*g*. during blooms). The numbers for *Prochlorococcus*, and probably for SAMs generally, are equally striking. With this kind of mutational saturation, there is no waiting time for the adaptive mutations to arise. Adaptation is expected to proceed quickly (with timescales of months to years), and to result from the spread of adaptive alleles that arise many times independently (Figure 1). This process has been seen during the evolution of insecticide resistance in Drosophila [47]. Given that populations of SAMs are many orders of magnitude larger, this evolutionary regime is likely the predominant way that adaptation works in SAMs. Whether this is the case could be tested by analysing patterns of molecular variation, as discussed below.

### Recombination, linkage disequilibrium, and population size

The key evolutionary effect of recombination is to break down non-random associations (linkage disequilibrium, LD) between alleles at different loci. This can have a profound effect on adaptation: decreased LD allows alleles at different loci to evolve independently [48]. Consider this extreme case: in a population without recombination, allele A_1_ at locus A always occurs with allele B_1_ at locus B. Then selection that causes A_1_ to spread to fixation will also cause fixation of B_1_ even if that allele is completely free of selection. If loci A and B both experience selection, “selective interference” occurs, and neither adapts as quickly as it would in the absence of the other. Selective interference can be particularly strong in asexual species, where LD builds up across the entire genome. Further, the strength of selective interference grows with population size.

Blooms of phytoplankton, such as coccolithophore *G. huxleyi*, are thought to be dominated by the rapid growth of multiple clones that mainly reproduce asexually [49] (though, sex during blooms has been reported, e.g. in diatoms [50,51]). It is therefore surprising that *G. huxleyi* has extremely low LD [38]. How can we reconcile frequent clonal reproduction and low LD? The extent of LD depends on how much recombination occurs in the population, which is measured by population-scaled recombination rate (ρ). As ρ (= 4*N*_e_ *r*) depends on the product of per-individual recombination rate (*r*) and the effective population size (*N*_e_), in very large populations ρ can be large (and LD small) even if *r* is low. Thus, even if sexual reproduction is infrequent, there is enough recombination in a very large population to break down LD. This means that even in the SAM species with relatively rare sexual reproduction, LD is likely low and even the sites at short distances from each other are independent in evolutionary sense, with relatively little selective interference occurring. Low LD and selective interference in SAM genomes ensure higher efficacy of selection, which should help their adaptation.

## Are the current evolutionary genetic approaches applicable to SAMs?

Many of the evolutionary genetic models (e.g.[52]) assume infinitely large populations, which is a reasonable approximation for very large SAM populations. However, as discussed above, SAMs likely violate the assumptions of the nearly neutral theory [46] which serves as a foundation for many of the widely used evolutionary genetic approaches that can be problematic for SAMs. A simple (but highly conservative) statistical test for adaptation at a gene compares the rate of substitution at silent (synonymous) sites, which is denoted as *K*_s_ (or *D*_s_), with the rate at nonsilent (nonsynonymous) sites, denoted as *K*_a_ (or *D*_n_) [53]. One typically assumes that non-silent sites evolve under selection, while silent sites are evolving neutrally, which is likely incorrect for extremely large populations of SAM, as discussed above. Most often, selection is purifying, which decreases the substitution rate at nonsilent sites, and so the *K*_a_/*K*_s_ is less than 1. On the other hand, when positive selection (adaptation) does occur at nonsynonymous sites, it increases their substitution rates. A *K*_a_/*K*_s_ ratio that exceeds 1 is therefore taken as evidence of adaptation at a gene [53]. But what if silent sites do in fact experience selection? The prediction is then less clear. A plausible argument is that selection (both purifying and positive) will generally be weaker on silent than on nonsilent sites. If so, then *K*_a_/*K*_s_ > 1 would again suggest adaptation is occurring at the nonsilent sites. The HKA [54] and the MK [55] tests to detect selection in DNA sequence data are more powerful than the *K*_a_/*K*_s_ ratio, but they are also more sensitive to violations of the assumption that silent sites evolve neutrally. The DFE-alpha method [56], which estimates the fitness effects of new mutations and the fraction of substitutions caused by selection and by drift, will likewise fail if no sites in the genome are evolving neutrally. The suitability of these approaches for SAMs is questionable, but the use of pseudogenes as a neutral reference [20] may make these approaches applicable to SAMs if mutations in pseudogenes are neutral.

Another theory-related problem is that SAMs may violate the assumptions of the “coalescent” theory [53,57] that is the foundation for many of our inferences in evolutionary genetics (Box 3). A key assumption in this framework is that the evolutionary histories of genes (genealogies) are bifurcating trees. However, this assumption is likely to be violated in many marine organisms [58], including SAMs, where seasonal phytoplankton blooms can be dominated by a few actively reproducing clones [49]. This may cause multiple branches in the genealogy to descend from a single ancestor, a highly successful clone that disproportionately contributed to future generations. This would violate the classical coalescent model, resulting in more “star-like” gene genealogies with shorter internal branches (Box 3), decreased neutral diversity (π), and altered distributions of allele frequencies. Among the consequences are that standard statistics to estimate demographic history may fail. Tajima’s *D* [59] is a widely-used statistic that is based on the distribution of allele frequencies. A negative value of *D* is often taken as evidence of recent population growth [59], but negative values can also result from multiple mergers in gene genealogies [60]. The good news here is that alternatives to the standard coalescent model (“multiple merger coalescent” [MMC] models) are being developed that could be appropriate to SAMs [58,60]. An application of MMC in microbial population genetics revealed that previous conclusions based on standard coalescent process may need to be revised [61].

The very large populations of SAMs confront phylogenetic reconstruction with two difficulties. The first is “incomplete lineage sorting” (ILS), which occurs when the times between successive nodes on a gene tree (the “coalescent events”) are greater than the times between phylogenetic branching events (speciation) [62]. The result of ILS is that the tree inferred from a gene will often be incongruent with the true phylogenetic relationship in the species tree. As the extent of ILS is proportional to the population size at the time of speciation (see Figure 4 in [63]), ILS is expected to be extensive in SAMs, unless speciation is associated with a population bottleneck, for example when a new species forms in a small lagoon cut off from the sea or via a genome rearrangement, such as polyploidisation, that creates a reproductive barrier [13]. The second difficulty is “mutational saturation”, which blurs the phylogenetic signal when recurrent mutations occur independently in different lineages, is expected to occur in very large populations when *N*_e_ μ >> 1 [64]. In principle, phylogenetic methods based on appropriate assumptions [65] can accommodate both ILS and mutational saturation, but the phylogenetic signal may be weaker than for species with modest population sizes. Recently, methods have been proposed that use genome sequences to delimit species boundaries that are explicitly suited to SAMs [64]. They do, however, forfeit the goal of finding the phylogenetic relations between the species.

## What approaches can we use to study the evolution of SAMs?

Microbial evolution is often studied in microcosm [66,67] or reciprocal transplant [33] experiments in the lab. However, lab-based microcosms have limited capacity and even semi-natural mesocosm experiments [68] can only accommodate population sizes that are many orders of magnitude smaller than natural SAM populations in world oceans. Given the importance of population size for the ways evolution works (discussed above), it is important to study evolutionary processes in natural SAM populations. Below we discuss what evolutionary genetic approaches are suitable for this purpose and what tools are likely to fail in astronomically large SAM populations.

We have seen that superabundant microbes will challenge many of the evolutionary genetic methods, but some of the existing approaches may be useful and accurate. Intuitively, linkage disequilibrium-based (LD) statistics, such as Z_nS_ [69], and the statistics based on allele frequency, such as Tajima’s *D* [59], may be suitable for SAMs. These applications would, however, require care in choice of the appropriate null models, perhaps replacing standard coalescent with MMC (as discussed above), or using an empirical distribution of the statistic across the genome. The allele-frequency-based analyses are informative about population structure, past species demography and selective pressures (e.g. [23]). Demographic inferences based on allele frequency distributions (e.g. [70]) may also be used in SAMs with models that account for the non-neutrality of most polymorphisms in very large populations. Such approaches are useful to study past population and species dynamics, infer population size changes through time and estimate the rate of interspecific gene flow [24,26–28] after speciation. This can be very informative about the ways new species form in SAMs [13].

Other evolutionary genetic approaches based on allele frequencies can also be adapted for the analysis of SAM data. Clines, which are smooth spatial gradients in allele frequencies or phenotypes, can form when loci adapt to environmental gradients [71]. Many marine plankton show clines associated with latitude, depth, salinity, and other environmental variables [72–74]. The spatial form of these clines could be used to estimate important evolutionary quantities such as how rapidly selection varies in space and the rates of movement between populations [75,76]. It is worth noting that the deterministic models of clines [52] assume infinite population size, which is a reasonable approximation for SAMs. The genome-wide analysis of clines in SAMs could reveal the number of loci adapted to local environmental conditions. Correlations between phenotypic traits, environmental conditions, and allele frequencies could identify the genes contributing to locally adapted phenotypes [23,77].

Codon bias occurs when different codons that correspond to the same amino acid occur at unequal frequencies across the genome. The classical explanation, which involves very weak selection acting on these synonymous (silent) alleles, is expected to produce stronger codon bias in larger populations as selection becomes more powerful relative to drift [78]. The recent discovery that phytoplankton species do not show very strong codon bias therefore came as an intriguing surprise [79]. These results may point to alternative hypotheses for codon bias [80–82]. The vast population sizes of SAMs provide unique opportunities to test these ideas.

Over the last decade, a major goal of evolutionary genomics has been to identify regions of the genome involved in recent adaptation. One approach that is widely used for this purpose is to search for regions of the genome that were depleted of diversity as beneficial mutations spread to fixation (Figure 1). Parameter-free approaches (e.g. [83]) could be used to detect selective sweeps in SAMs. But the extremely large population sizes may make sweeps of this sort very rare. As discussed above, in marine phytoplankton and other species with populations so large that *N*μ >> 1, adaptive mutations are likely to arise many times independently (Figure 1). In that case, windows of low diversity are not expected to form in populations that are roughly constant in size [84] – a prediction that can be tested in SAMs.

Another useful application of DNA polymorphism data analysis is to estimate rates of recombination, sexual reproduction and self-fertilization in natural populations [16,18,85–88]. Some of the statistical methods developed for this purpose rely on the assumption of neutral evolution at silent sites [87] so in their present form they may be inappropriate for use with SAMs. Instead, it may be safer to use heuristic methods to detect recombination in superabundant microbes (e.g.[88]) as they do not depend on explicit models of evolution. The relationship between population-scaled recombination rate (ρ) and *N*_e_ mentioned above (ρ = 4*N*_e_ *r*) provides a way to estimate effective population size from LD [89] independently from Eq. (1) in Box 1, but this has not yet been done for any SAM species.

A very different perspective on the evolution of SAMs would come from studies that track allele frequency changes in time. These time series can be used to directly measure genetic drift in real time, and the so-called “variance effective population size” [90]. Unlike the estimates of *N*_e_ from Eq. (1), which averages over long time periods, this approach yields estimates of the current *N*_e_ that are unaffected by population bottlenecks in the past. Selection results in time series that look quite different than those caused by drift: it produces consistent directional changes in allele frequencies. Thus, time series are able to parse out the contributions of drift and selection to evolutionary change. This sort of analysis has been done with bacterial populations in the lab [67,91], but has not been attempted for any free-living marine microorganism. Even time series sampled over just a few years may be sufficiently long to study adaptation in SAMs, as they can go through many generations per year. Such analyses would be informative about the timescale required for adaptation in SAMs to occur – is it fast enough for SAMs to adapt to seasonal changes, or even to rapidly changing conditions during a single phytoplankton bloom? The largest and the longest (since 1931) long-term plankton sampling is conducted by the Continuous Plankton Recorder (CPR) survey [92], and the methods for high-throughput sequencing of formamide-preserved CPR samples are being developed [93]. Smaller scale time series plankton samples are also collected by various marine laboratories, but they are mainly used for metabarcoding to analyse species richness and its temporal variation [94,95]. Wider use of these serial samples for whole genome metagenomic sequencing would answer many questions regarding SAM evolution discussed above.

The fossil record that is available for some abundant marine plankton can provide a perspective that complements inferences drawn from molecular data. The calcium carbonate shells of coccolithophores and foraminifera are well preserved in the fossil record and can be used to estimate relative abundance through time. A recent study of the coccolithophore genus *Gephyrocapsa* revealed a good correspondence between genetic estimates of population size change through time and species abundance in the fossil data (Figure 2 D-F in [24]). Such integrated evolutionary genetic and palaeontological analyses provide a way to cross-validate the two independent lines of evidence, each of which has its own strengths and weaknesses.

## Concluding remarks

Microevolutionary processes are the very foundation of evolutionary change, yet they remain woefully understudied in superabundant microbes, including many species of marine plankton [7,8]. Population size is one of the most important parameters in evolutionary genetics [71,90], and precisely because of their astronomical abundance microevolution in SAMs may work in rather unusual ways [7,12,38]. We suggest that evolution in SAMs may conform to the panselectionist view that dominated in biology prior to the current era of the neutral and nearly neutral [46] theories. Testing this idea and (more generally) studying how microevolution works in SAMs will require new evolutionary genetic approaches suitable for astronomically large populations.

## Box 1 Drift, population size, and genetic diversity

Population size determines the number of new mutations occurring in the population and the rate of loss of mutations by random genetic drift – stochastic variation in allele frequency over generations. Drift, which is stronger in smaller populations and weaker in larger ones, is typically quantified by the “effective population size”, symbolised as *N*_e_ [71,90]. While there are several definitions of *N*_e_ [90], for our purposes it can be thought of as the size of an imaginary ‘ideal’ population that has allele frequency fluctuations as large as those in the population of interest. The size of the ideal population is constant, and all individuals make equal contributions to the next generation [71].

The larger the population, the more genes mutate every generation, increasing the influx of genetic diversity. These mutations experience genetic drift and can be lost by chance, which limits overall genetic diversity in the population. Genetic diversity (π) is defined as the chance that two copies of a gene randomly chosen from a population will carry a different nucleotide at a given site. At a site in the genome that is free of selection and in a population of constant size, the balance between the influx of new mutations and their loss by genetic drift is determined by the product of the effective population size (*N*_e_) and the mutation rate (μ):

(1) $$\text{π}=4N_{\text{e}}\text{μ}.$$

The factor of 4 in Eq. (1) pertains to diploids, and is replaced by a factor of 2 for haploids.

## Box 2 Population size and selection

Unlike drift, selection causes systematic changes in allele frequencies, with positive selection increasing the frequencies of adaptive alleles and negative selection reducing frequencies of deleterious alleles. The strength of selection is quantified by the “selection coefficient” (*s*). This quantity is the proportional increase or decrease that the mutation makes to an individual’s “fitness”, that is, the number of offspring it expects to contribute to the next generation. The selection coefficient is negative when the mutation is deleterious and positive when advantageous. When a mutation has no effect whatever on fitness, then *s* = 0, and we say it is “neutral”. But, as the nearly neutral theory [46] tells us, a mutation with non-zero *s* can evolve as if neutral when |*N*_e_ *s*| < 1 because its change in frequency over time is dominated by drift. The extremely large values of *N*_e_ in SAMs require *s* to be extremely close to zero to keep |*N*_e_ *s*| < 1, which may mean that none of their mutations evolve as if neutral. This situation may have no parallel in the vast majority of species on Earth.

## Box 3 Use of coalescent theory in experimental evolutionary genetic studies

Coalescent theory [53,71] can be used to create the null expectation for the patterns of polymorphism under a certain demographic scenario. For this purpose, multiple random gene trees (genealogies) are generated (Figure I, A-C), and mutations randomly added to them according to a set of rules from coalescent theory [57]. This creates a set of simulated datasets of the same size and level of polymorphism as the real dataset. Statistics such as π and Tajima’s *D* [59] are calculated for the simulated and the observed datasets. If the value of a statistic in the observed data falls in the tail of the distribution obtained from the simulated datasets (panel D in Figure I), the real sample significantly deviates from the null hypothesis. For example, in a stable population (A) the distribution of the statistic is centred around zero, while in an expanding population (B) it is shifted to negative values (D). The multiple merger coalescent (C) makes different predictions compared to the standard coalescent (D), which can lead to mis-inference. For example, a stable population evolving with multifurcating genealogies can be mistaken for an expanding population if the standard coalescent is used instead of MMC.
